# Crohn’s disease may promote inflammation in IgA nephropathy: a case–control study of patients undergoing kidney biopsy

**DOI:** 10.1007/s00428-022-03373-w

**Published:** 2022-07-09

**Authors:** Minako Akiyama, Kosuke Shimomura, Hiroshi Yoshimoto, Minako Sako, Makoto Kodama, Keiko Abe, Mariko Gunji, Dedong Kang, Takashi Takaki, Yukihiro Wada, Masayuki Iyoda, Kazuho Honda

**Affiliations:** 1 Department of Nephrology, JCHO Tokyo Yamate Medical Center, Tokyo, Japan; 2 Department of Anatomy, Showa University School of Medicine, 1-5-8 Hatanodai, Shinagawa-ku, Tokyo, 142-8555 Japan; 3 Department of Inflammatory Bowel Disease (IBD), JCHO Tokyo Yamate Medical Center, Tokyo, Japan; 4 Department of Pathology, JCHO Tokyo Yamate Medical Center, Tokyo, Japan; 5 Electron Microscopy Center, Showa University School of Medicine, Tokyo, Japan; 6 Division of Nephrology, Department of Medicine, Showa University School of Medicine, Tokyo, Japan; 7 Department of Microbiology and Immunology, Showa University School of Medicine, Tokyo, Japan

**Keywords:** Crohn’s disease, Inflammatory bowel disease, IgA nephropathy, Intestinal immunity, Galactose-deficient IgA1, IgA subclass

## Abstract

Intestinal immunity has been closely associated with the pathogenesis and progression of renal diseases, a relationship known as the “gut–kidney axis.” To determine the association between immunoglobulin A nephropathy (IgAN) and Crohn’s disease (CD), a clinico-pathological study was performed on patients who had IgAN with CD (CD-IgAN) and without CD (NOS-IgAN). We enrolled 29 patients diagnosed with IgAN via renal biopsy at the Tokyo Yamate Medical Center from 2009 to 2017. The patients were divided into CD-IgAN (*n* = 18) and NOS-IgAN (*n* = 11) and evaluated for clinical and pathological findings. IgA subclasses and galactose-deficient IgA1 (Gd-IgA1) were examined via immunohistochemistry using formalin-fixed paraffin-embedded sections from renal biopsy. Our results showed no significant difference in the extent of mesangial IgA subclasses or Gd-IgA1 deposition according to the presence or absence of CD. Pathologically, however, those with CD-IgAN had remarkably higher percentage of global glomerulosclerosis and extent of interstitial fibrosis and tubular atrophy (IF/TA) compared to those with NOS-IgAN. Moreover, the extent of macrophage infiltration in the glomerulus and interstitium was significantly higher in CD-IgAN than in NOS-IgAN. Clinically, the CD-IgAN group had significantly worse responsiveness to steroid treatment compared to the NOS-IgAN group. In conclusion, the similar immunological characteristics of deposited IgA molecules in the glomeruli between the CD-IgAN and NOS-IgAN groups might suggest their etiological similarity. However, a renal pathology showing advanced glomerular and tubulointerstitial sclerosis accompanying increased macrophage infiltration and highly resistant clinical features in patients with CD-IgAN suggests that some pathophysiological factors in CD, including abnormal intestinal immunity, may promote and activate the inflammatory process in IgAN via undetermined mechanisms.

**Supplementary Information:**

The online version contains supplementary material available at 10.1007/s00428-022-03373-w.

## Introduction

Recent studies have highlighted the importance of the gut–kidney axis and the involvement of inflammatory bowel disease (IBD) in renal disease [1]. Several studies on renal biopsy specimens from patients with IBD have reported complications of immunoglobulin A nephropathy (IgAN) [2, 3].

A study of 83 patients with IBD by Josephine et al. showed that IgAN was the most frequent complication (20 cases) [4]. Other studies have also investigated the relationship between CD and IgAN [5, 6]. Immunoglobulin A (IgA) has been suggested to play a major role in intestinal immunity because it is produced by IgA-producing B/plasma cells and secreted into the intestinal lumen through the intestinal epithelium. By contrast, studies have suggested that IBD, such as Crohn’s disease (CD) and ulcerative colitis, which have been associated with intestinal immune disorders, impairs IgA production and secretion. However, the precise B cell abnormalities underlying IgAN have yet to be elucidated [7]. Based on the aforementioned findings, the US NEFIGAN study reported that budesonide, a CD therapeutic and intestinal steroid, can be used to treat patients with IgAN and ameliorated proteinuria [8] [9]. Moreover, another study showed that budesonide was effective for IgAN that recurred after kidney transplantation [10]. Budesonide targets the Peyer’s patches of the intestine and is thought to suppress abnormal T-cell immunity, suggesting that modifying the intestinal immunity might result in renal disease.

IgAN, which is characterized by the deposition of IgAN immune complex in the mesangial region of the glomerulus, can be classified as idiopathic IgAN or secondary IgAN associated with infectious and liver diseases. In patients with idiopathic IgAN, the galactose-deficient IgA1 (Gd-IgA1) has a structure in which galactose is deficient in the O-linked sugar chain at IgA1 hinge and N-acetyl galactosamine (GalNAC) is exposed. Reports have shown that IgG immune complexes against this abnormal IgA1 are deposited in the glomerulus [11] [12]. In fact, the IgA subclass deposited in the glomerulus of most patients with idiopathic IgAN mainly include IgA1, with the quantity of IgA2 co-deposition being negligible. The increased level of serum Gd-IgA1 in IgAN [13], as well as the significant correlation between the extent of mesangial Gd-IgA1 deposition and clinical disease activity of IgAN, suggests the etiological role of Gd-IgA1 in IgAN [14]. However, recent evidence has shown that secondary IgAN, which occurs in various disease, can also be attributed to the deposition of Gd-IgA1 in the mesangial region [15]. Therefore, Gd-IgA1 may not be specific to primary IgAN, suggesting a similar immunological mechanism between secondary and primary IgAN [16].

To elucidate the pathogenetic relationship between IgAN and CD, we compared the clinical and histological findings of patients diagnosed with CD prior to IgAN (CD-IgAN) and those who had IgAN without CD (NOS-IgAN). In particular, we analyzed the IgA subclass composition, which is thought to be involved in the pathogenic mechanism of IgAN, the presence or absence of Gd-IgA1, and the degree of deposition.

## Materials and methods

### Patients and study design

We enrolled 29 adult patients (≥ 18 years old) who were diagnosed with IgAN based on the findings of renal biopsy specimens obtained between January 2009 and December 2017 at Tokyo Yamate Medical Center. The included patients were categorized into the following two groups: (1) CD-IgAN (*n* = 18) and (2) NOS-IgA (*n* = 11). Consecutive patients in the NOS-IgAN group were selected during the same period when patients in the CD-IgAN group were enrolled. The patients were not age or sex matched when selecting the two study groups. No selection bias was implemented during the enrollment to the NOS-IgAN group. The higher number of patients with CD-IgAN than those with NOS-IgAN was because our hospital specializes in treatment of inflammatory bowel diseases, and the male predominance in CD-IgAN group was caused by the male predominance of IBD in our hospital. The clinical and histopathological characteristics were examined.

IgAN was pathologically diagnosed through glomerular IgA deposition and C3 deposition determined via immunofluorescent microscopy (IF). Moreover, clinical information was collected. Patients whose renal biopsy specimens had insufficient sample volume or contained no glomerulus were excluded from the study. When more than one renal biopsy was performed during the observation period, the first biopsy sample was selected for histological evaluation.

For the diagnosis of CD, we included individuals whose diagnosis was confirmed by our gastroenterologists based on clinical, endoscopic, and histopathological findings of colorectal specimens before the renal biopsy.

All patients provided informed consent at the time of renal biopsy and were able to opt-out from the study if they desired. This study was approved by the Ethics Committee of the Tokyo Yamate Medical Center (J023) and conducted in accordance with the ethical standards enshrined in the Declaration of Helsinki.

### Clinical parameters

The following variables patient characteristics were retrospectively obtained from the medical records: age, gender, duration from the disease onset to renal biopsy (years), body mass index (BMI) (kg/m^2^), body weight (kg), systolic blood pressure (mmHg), use of renin–angiotensin system inhibitors (RASI), serum creatinine (Cr) level (mg/dL), serum IgA level (mg/dL), hematuria, and proteinuria. Hematuria and proteinuria (before and after treatment) were scored semi-quantitatively based on the urine test strip method. The grade was determined as 0, 0.5, 1, 2, and 3 when the result was ( −), ( ±), (1 +), (2 +), and (3 +) on the strip test, respectively. Treatments for IgAN were divided into oral steroids, intravenous steroid pulse (IVP), tonsillectomy, and their combination, such as oral steroid therapy and tonsillectomy. The number of patients administered 5-aminosalicylic acid (5-ASA) was only counted in those with CD-IgAN. To evaluate therapeutic response, we compared the number of patients with hematuria (score ≥ 1) and proteinuria (score ≥ 1) before and after the treatment in the CD-IgAN and NOS-IgAN groups.

### Histopathological assessment

Histopathologic findings were independently reviewed by a renal pathologist (K.H.) and nephrologist (M.A.) who were blinded to the clinical background of each section. The rate of global glomerular sclerosis was defined as the percentage of globally sclerotic glomerulus among all glomeruli in one section. The rate of active phase crescent was defined as the rate of cellular or fibrocellular crescent among all glomeruli in one section. Furthermore, we evaluated the histological scoring, MEST-C, using the Oxford’s classification for IgAN [17, 18].

We performed periodic acid–Schiff stain and Masson trichrome stain (Masson) and classified the extent of interstitial fibrosis and tubular atrophy (IF/TA) into five grades according to the percentage of affected area in the total cortical area: 0 (0–5%), 1 (6–25%), 2 (26–50%), 3 (51–75%), and 4 (76–100%). The extent of interstitial inflammatory cell infiltration was also evaluated and semi-quantitatively classified into four grades: 0 (absent), 1 (mild), 2 (moderate), and 3 (severe). Vascular lesions were evaluated based on the degree of intimal hyalinosis and luminal narrowing in the arterioles and degree of intimal thickening accompanied by the proliferation of elastic fibers and luminal narrowing in the interlobular arteries. Arteriolar hyalinosis and interlobular arteriosclerosis were graded as 0 (absent intimal lesion), 1 (mild intimal lesion without luminal narrowing), 2 (moderate intimal lesion with unremarkable luminal narrowing), and 3 (severe intimal lesion with remarkable luminal narrowing) depending on the severity of each lesion.

To compensate for the small cohort size of the NOS-IgAN group in this study, a meta-analytic comparison of MEST-C scores was performed using two large-scale IgAN cohorts included in previous studies in the literature [19, 20].

### Immunohistological assessment

The staining protocol for Gd-IgA1 was described in the previous literature [14], and glomerular Gd-IgA1 deposition was examined via immunohistochemistry (IHC). Briefly, deparaffined sections were heated with buffer (Histofine®, Nichirei, Tokyo, Japan) in an autoclave at 121 °C for 30 min for antigen retrieval. After endogenous peroxidase was quenched with 0.3% H_2_O_2_ in methanol, nonspecific binding was blocked using protein blocking solution, and the sections were incubated overnight at 4 °C with rat monoclonal anti-human Gd-IgA1 antibody (KM55) diluted to 1:100, followed by EnVision™ + Dual Link System-HRP (Dako, Glostrup, Denmark) for 60 min at room temperature. Diaminobenzidine (DAB) (Dako) was used for visualization.

The staining intensity of IgA (#AR045-5R, BioGenex), IgA1(#9130–01, Southern Biotech, 1:20,000 antibody dilution, activation time 15 min), IgA2 (#9140–01, Southern Biotech,1:10,000 antibody dilution, activation time 15 min), and Gd-IgA1 (KM55, #10,777, Immuno-Biological Laboratories) in each glomerulus was classified into four grades: 0 (negative), 1 (weak, 1 +), 2 (moderate, 2 +), and 3 (strong, 3 +). One pathological slide was assigned to each case, and all glomeruli in one slide were classified into four grades. All of glomeruli on each slide were assessed and categorized into four grades. Thereafter, staining intensity was determined as the summation of these grades divided by the total number of glomeruli on each slide. Immunofluorescent intensity of glomerular C3 deposition was evaluated and classified into four grades: 0 (negative), 1 (weak, 1 +), 2 (moderate, 2 +), and 3 (strong, 3 +) based on the descriptions in laboratory reports of routine immunofluorescent examination using frozen sections of the specimens.

We also evaluated glomerular and interstitial macrophage infiltration by immunohistochemistry using primary monoclonal antibody against CD68 (clone: PG-M1, DAKO, Glostrup, Denmark). For glomerular macrophage infiltration, the extent of glomerular CD68 ( +) cell infiltration was evaluated semi-quantitatively in the microscopic field at high magnification (× 400) and classified into four grades: 0 (none), 1 (mild, < 5 cells/glomerulus), 2 (moderate, 5–9 cells/glomerulus), and 3 (severe, ≥ 10 cells/glomerulus). For interstitial macrophage infiltration, the extent of interstitial CD68 ( +) cell infiltration was semi-quantitatively evaluated in the microscopic field at the middle magnification (× 200) and classified into four grades: 0 (none), 1 (mild), 2 (moderate), and 3 (severe).

### Statistical analysis

Data were expressed as the means ± *SD* or percentages (%). Nonparametric continuous variables were compared using Mann–Whitney *U* test. Categorical variables were compared using Fisher’s test and McNemar’s test. Meta-analytic comparison of the MEST-C scores using the cohorts included in previous literatures was performed using Fisher’s exact probability test. We also evaluated the correlation between disease duration of CD and IF/TA grade or global glomerular sclerosis rate using Spearman’s rank correlation coefficient and created scatter plots. All statistical analyses were performed using EZR (Saitama Medical Center, Jichi Medical University, Saitama, Japan), a graphical user interface for R (The R Foundation for Statistical Computing, Vienna, Austria), with *p* values < 0.05 indicating statistical significance.

## Results

### Clinical characteristics of patients

Table S1 shows the clinical background and laboratory findings of the 29 patients with IgAN studied, among whom 18 and 11 belonged to the CD-IgAN and NOS-IgAN groups, respectively. The mean serum creatinine level at renal biopsy was 1.5 (± 0.8) and 0.9 (± 0.3) mg/dL for the CD-IgAN and NOS-IgAN groups, respectively, with the former having significantly higher levels (*p* = 0.003). BMI was 20.8 (± 3.0) and 23.8 (± 3.3) kg/m^2^ for CD-IgAN and NOS-IgAN, respectively, with the former having significantly lower mean values (*p* = 0.012). Regarding sex, 17 (94.4%) and 6 (54.5%) subjects in the CD-IgAN and NOS-IgAN groups were males, with the former being more male predominant (*p* = 0.018). Aside from gender, serum creatinine level, and BMI, no significant differences between the two groups were noted in the following clinical backgrounds: duration from onset of urinary abnormality to biopsy, blood pressure, hematuria or proteinuria grade, serum IgA level, and therapeutic intervention for IgAN.

### *Comparison of histological findings between the CD-IgAN and NOS-IgAN groups (Table **1**)*

**Table 1:** Comparison of histopathologic findings between IgAN patients with and without Crohn’s disease

Characteristics	CD-IgAN (*n* = 18)	NOS-IgAN (*n* = 11)	*p-value*
	Mean ± *SD*	Mean ± *SD*	
Glomerular lesions			
Global glomerular sclerosis (%)	29.6 ± 31.3	5.1 ± 6.0	0.023*
Crescent in active (%)	7.5 ± 10.7	1.9 ± 3.4	NS (0.22)
Crescent in active (%) in patients treated with steroids	12.3 ± 11.1^a^	2.3 ± 3.7^b^	0.020*
Oxford classification score (MEST-C)			
M	0.44 ± 0.51	0.18 ± 0.40	NS (0.234)
E	0.06 ± 0.24	0.00 ± 0.00	NS (1.00)
S	0.22 ± 0.43	0.00 ± 0.00	NS (0.268)
T	0.56 ± 0.71	0.00 ± 0.00	0.046*
C	0.33 ± 0.49	0.18 ± 0.40	NS (0.671)
Tubulo-interstitial lesions			
IF/TA grade (grade)^c^	1.50 ± 0.99	0.36 ± 0.50	0.017*
Inflammatory cell infiltration (grade)^d^	1.17 ± 0.62	0.64 ± 0.50	NS (0.092)
Vascular sclerotic lesions			
Arteriolar hyalinosis (grade)^d^	0.61 ± 0.61	0.0 ± 0.0	0.003**
Interlobular arteriosclerosis (grade)^d,e^	0.21 ± 0.43 (*n* = 14)	0.25 ± 0.46 (*n* = 8)	NS (1.00)
Macrophage infiltration (by CD68 staining)			
Glomerular CD68( +) cell infiltration (grade)^f^	1.45 ± 0.51	1.13 ± 0.83	0.02*
Interstitial CD68( +) cell infiltration (grade)^g^	2.06 ± 0.68	1.13 ± 0.35	0.011*

Mann–Whitney *U* test or Fisher’s test were used for statistical analysis

Abbreviations: *IF/TA* interstitial fibrosis/tubular atrophy, *CD-IgAN* immunoglobulin A nephropathy patients with Crohn’s disease, *NOS-IgAN* immunoglobulin A nephropathy patients without Crohn’s disease, *NS* not significant

^*^p < 0.05; **p < 0.01

^a^Nine patients in the CD-IgAN group were treated with steroids

^b^Nine patients in the NOS-IgAN group were treated with steroid

^C^The grade of interstitial fibrosis and tubular atrophy (IF/TA) was classified into five based on the percentage of the total cortical area affected: 0 (0–5%), 1 (6–25%), 2 (26–50%), 3 (51–75%), and 4 (76–100%)

^d^The grade was classified into four grades: 0 (absent), 1 (mild), 2 (moderate), and 3 (severe)

^e^The interlobular artery was not detected in 4 and 3 patients in the CD-IgAN and NOS-IgAN groups, respectively

^f^The grade of glomerular CD68 ( +) cell infiltration was classified into four: 0 (none), 1 (mild, < 5 cells/glomerulus), 2 (moderate, 5–9 cells/glomerulus), and 3 (severe, ≥ 10 cells/glomerulus). CD68 immunohistochemical staining was performed in 16 and 8 patients in the CD-IgAN and NOS-IgAN groups, respectively

^g^The grade of interstitial CD68 ( +) cell infiltration was classified into four: 0 (none), 1 (mild), 2 (moderate), and 3 (severe)

**Fig. 1:**
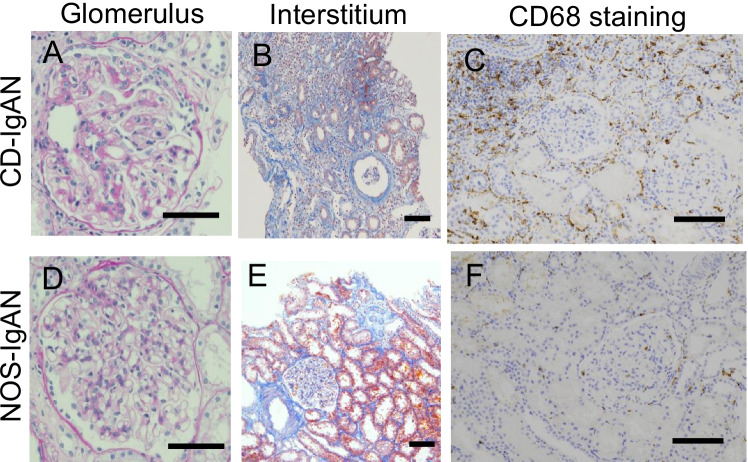
Typical histological findings and macrophage infiltration of the glomerulus and tubulointerstitium in the CD-IgAN and NOS-IgAN groups. The upper row shows the staining findings of CD-IgAN (**A**, **B**, and **C**) and the lower row shows the staining findings of NOS-IgAN (**D**, **E**, and **F**). Glomerular sclerosis and IF/TA are more severe in CD-IgAN than in NOS-IgAN. Glomerular and interstitial macrophage infiltration was observed with various degrees in both groups, depending on the histological alterations. (**A** and **D**, periodic acid–Schiff stain; **B** and **E**, Masson trichrome stain; **C** and **F**, CD68 immunohistochemical stain, bar = 50 μm)

Figure 1 shows the typical renal biopsy findings for the glomerulus and tubulointerstitium in the CD-IgAN and NOS-IgAN groups. Glomerular and interstitial macrophage infiltrations detected by anti-CD68 immunohistochemical staining are also presented. Cases with CD-IgAN had more prominent sclerosing glomerular lesions compared to those with NOS-IgAN. CD-IgAN had more extensive and severe fibrosis than NOS-IgAN. Glomerular and interstitial macrophage infiltrations were observed in both patients depending on glomerular and interstitial lesions.

The differences in histopathological findings between the 18 CD-IgAN cases and 11 NOS-IgAN cases are quantified and compared in Table 1. The mean global glomerular sclerosis rate was 29.6% (± 31.3) and 5.1% (± 6.0) in the CD-IgAN and NOS-IgAN groups, with the former showing a significantly higher rate (*p* = 0.023). The mean proportion of glomeruli containing active crescents was 7.5% (± 10.7) and 1.9% (± 3.4) for the CD-IgAN and NOS-IgAN groups, with no significant differences between both groups (*p* = 0.22), whereas in patients subsequently treated with steroids, it was significantly higher in the CD-IgA group (*n* = 9) than in the NOS-IgAN group (*n* = 9) (12.3% [± 11.1] and 2.3% [± 3.7], respectively [*p* = 0.02]). Among the MEST-C scores defined via the Oxford classification, T score was significantly higher in CD-IgAN group than in NOS-IgAN group (0.56 ± 0.71, 0 ± 0, *p* = 0.046), whereas the other scores including M, E, S, and C did not show any significant differences between the CD-IgAN and NOS-IgAN groups.

Concerning tubulo-interstitial lesions, none of the patients with NOS-IgA showed interstitial fibrosis/tubular atrophy (IF/TA) grade 2 or higher lesions, whereas 9 out of 18 cases (50%) with CD-IgAN had grade 2 or higher lesions, suggesting that the CD-IgA group had significantly higher IF/TA grade compared to the NOS-IgAN group (1.50 ± 0.99 vs. 0.36 ± 0.50, *p* = 0.017), whereas the extent of interstitial inflammatory cell infiltration tended to be higher in the CD-IgAN group than in the NOS-IgAN group but not significantly different (1.17 ± 0.62 vs. 0.64 ± 0.50, *p* = 0.092).

The comparison of vascular lesions yielded the following results. Arteriolar hyalinosis was observed in 44% of cases with CD-IgAN but none of those with NOS-IgAN (*p* = 0.012). Moreover, interlobular arteriosclerosis was detected in 21% of the patients with CD-IgAN and but none of those with NOS-IgAN (*p* = 0.273), although the difference was not statistically significant. The average grade of arteriolar hyalinosis was 0.61 ± 0.61 and 0 ± 0 in the CD-IgAN and NOS-IgAN groups, with the former showing a significantly higher grate (*p* = 0.003). However, the average grade of interlobular arteriosclerosis was not different between CD-IgAN and NOS-IgAN groups.

The extent of glomerular macrophage infiltration was significantly higher in CD-IgAN group than in NOS-IgAN group (1.45 ± 0.51 vs. 1.13 ± 0.83, *p* = 0.02). The extent of interstitial macrophage infiltration was also significantly higher in CD-IgAN group than in NOS-IgAN group (2.06 ± 0.68 vs. 1.13 ± 0.35, *p* = 0.011).

Meta-analytic comparisons of the MEST-C scores between the CD-IgAN group of the present study and the IgAN cohorts of the previous studies [19, 20] revealed that the incidence of T1/T2 was significantly higher in our CD-IgAN group than in Barbour’s IgAN cohort [19] (44.4% vs. 22.1%, *p* = 0.045) and Kamano’s cohort [20] (44.4% vs. 11.4%, *p* < 0.001). Conversely, the incidence of S1 was significantly lower in our CD-IgAN group than in Barbour’s IgAN cohort (22.2% vs. 75.0%, *p* < 0.001) and Kamano’s cohort (22.2% vs. 79.0%, *p* < 0.001). Additionally, the incidences of E1 and C1/2 in our CD-IgAN group were significantly lower than those in Kamano’s cohort (E1: 5.6% vs. 35.3%, *p* = 0.0098, C1/2: 33.3% vs. 59.1%, *p* = 0.005) but did not significantly differ from those in Barbour’s cohort (Table S3). Furthermore, the comparisons of MEST-C scores between our NOS-IgA group and these two cohorts revealed that the incidence of S1 was significantly lower in our NOS-IgA group than in both cohorts (0% vs.75.0% and 79.0%, *p* < 0.001). The incidences of E1 and C1/2 in our NOS-IgA group were also significantly lower than those in Kamano’s cohorts (E1: 0% vs. 35.3%, *p* = 0.011 and C1/2: 18.2% vs. 59.1%, *p* = 0.010) but did not differ from those in Barbour’s cohort. No significant difference in M and T scores was observed between our NOS-IgAN cohort and the two large-scale IgAN cohorts (Table S4).

### No significant correlation between Crohn’s disease duration and global glomerular sclerosis or IF/TA

Figure S1 shows the correlation between CD duration and global glomerular sclerosis (A) or IF/TA (B). No significant correlation was observed between global glomerular sclerosis and the duration of CD (Spearman’s rank correlation coefficient 0.39, *p* = 0.11). Similarly, no significant correlation was observed between IF/TA grade and the duration of CD (Spearman’s rank correlation coefficient 0.45, *p* = 0.060). Moreover, the extents of arteriolar hyalinosis and interlobular arteriosclerosis were not correlated with the duration of CD (data not shown).

### Comparison of immunostaining results for IgA, C3, IgA subclass, and Gd-IgA1 between the CD-IgAN and NOS-IgAN groups

IgA subclass comprises IgA1 and IgA2, and representative image of each staining grade is shown in Figure S2. IgA1 and IgA2 were respectively deposited specifically in the mesangial region. No difference in deposition site of IgA subclass was observed regardless of the presence or absence of CD. The extent of IgA, IgA1, IgA2, and Gd-IgA1 deposition was represented as the average staining grade of all glomeruli on each specimen and compared between the CD-IgAN and NOS-IgAN groups (Table 2). Although the staining intensity of IgA, IgA1, and IgA2 was generally higher in NOS-IgAN than in CD-IgAN, a statistical significance was demonstrated only in IgA1 (1.83 ± 0.86 vs. 2.66 ± 0.80, *p* = 0.029) but not in IgA (2.26 ± 0.67 vs. 2.49 ± 0.42, *p* = 0.52) and IgA2 (1.39 ± 0.86 vs. 1.77 ± 0.26, *p* = 0.155) according to the presence and absence of CD complication. Moreover, no significant difference in the intensity ratio of IgA2/IgA1 was found between the two groups (0.78 ± 0.27 vs. 0.70 ± 0.26, *p* = 0.469).

**Table 2:** Comparison of immunohistochemical intensity of glomerular IgA, IgA subclass, Gd-IgA1, and C3 deposition between IgAN patients with and without Crohn’s disease

Glomerular deposition by immunohistochemistry	CD-IgAN (*n* = 18)	NOS-IgAN (*n* = 11)	*p-value*
	Mean ± *SD*	Mean ± *SD*	
IgA	2.26 ± 0.67	2.49 ± 0.42	NS (0.520)
IgA1	1.83 ± 0.86	2.66 ± 0.80	0.029*
IgA2	1.39 ± 0.86	1.77 ± 0.64	NS (0.155)
IgA2/IgA1	0.78 ± 0.27	0.70 ± 0.26	NS (0.469)
Galactose-deficient IgA1	1.17 ± 0.79	1.52 ± 0.65	NS (0.240)
C3^a^	1.57 ± 1.02	2.22 ± 0.67	NS (0.543)

Mann–Whitney *U* test or Fisher’s test was used for statistical analysis. Values were expressed as mean ± *SD*. The grade of each patient was obtained by dividing the total of the deposition grade of IgA, IgA1, IgA2, and Gd-IgA1 in each glomerulus by the total number of glomeruli

Abbreviations: *CD-IgAN* immunoglobulin A nephropathy patients with Crohn’s disease, *NOS-IgAN* immunoglobulin A nephropathy patients without Crohn’s disease, *NS* not significant

^*^p < 0.05

^a^The grade of C3 deposition was determined by performing routine immunofluorescent examination using frozen section and classified into 4 grades: 0 (non), 1 (mild), 2 (moderate), and 3 (severe). C3 deposition could not be evaluated in 4 and 2 patients in the CD-IgAN and NOS-IgAN groups, respectively

**Fig. 2:**
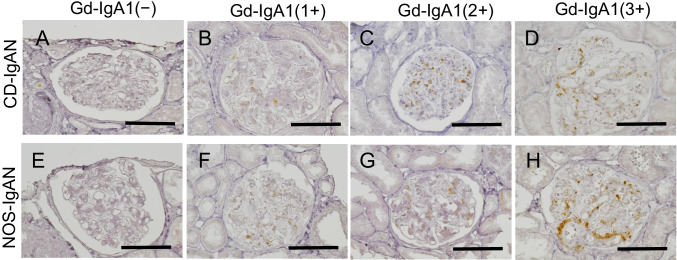
Typical Gd-IgA1 staining in glomeruli in the CD-IgAN and NOS-IgAN groups. The upper row shows the staining findings of CD-IgAN (**A**, **B**, **C**, and **D**), and the lower row shows the staining findings of NOS-IgAN (**E**, **F**, **G**, and **H**). From left to right, glomeruli with negative, + 1, + 2, and + 3 staining intensities are shown (× 400; bar = 50 μm). There was no difference in the deposition site and morphology of Gd-IgA1 due to the presence or absence of CD

Thereafter, the typical glomerular depositions of Gd-IgA1 staining were assessed separately for the CD-IgAN and NOS-IgAN groups (Fig. 2). Gd-IgA1 was specifically deposited in the mesangial region, with no significant difference according to the presence and absence of CD complication, similar to the IgA subclass. The extent of Gd-IgA1 deposition was evaluated by similar manner performed in IgA subclass evaluation and compared between the CD-IgAN and the NOS-IgAN groups (Table 2). No significant difference in the staining intensity was observed according to the presence or absence of CD complication (1.17 ± 0.79 vs.1.52 ± 0.65, *p* = 0.240). The grade of C3 deposition was semi-quantitatively evaluated by routine immunofluorescent examination for renal biopsy diagnosis. Although 3 of the 14 CD-IgAN patients (21.4%) and none of the 9 NOS-IgAN patients (0%) were negative for C3 deposition, the mean intensity grade of C3 deposition was not different statistically between the CD-IgAN and the NOS-IgAN groups (1.57 ± 1.02 vs. 2.22 ± 0.67, *p* = 0.543).

### Comparison of steroid treatment effects between the CD-IgAN and NOS-IgAN groups

Figure S3 presents the changes in hematuria and proteinuria grade before and after steroid treatment only for targeting treated cases classified according to the presence or absence of CD. In the NOS-IgAN group, treatment significantly ameliorated both hematuria and proteinuria (*p* = 0.041 and 0.041, respectively). In the CD-IgAN group, however, poor amelioration of both hematuria and proteinuria was observed.

**Fig. 3:**
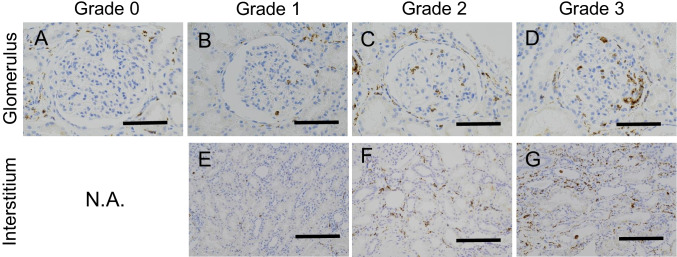
Histological grading of glomerular and interstitial macrophage infiltration. The upper row shows the CD68 staining findings of glomerulus (**A**, **B**, **C**, and **D**), and the lower row shows the CD68 staining findings of tubulointerstitium (**E**, **F**, and **G**). From left to right, glomerular and interstitial CD68 ( +) cell infiltrations with grades 0, 1, 2, and 3 of staining intensities are shown. Glomerular macrophage infiltration was classified into four grades: 0 (none), 1 (mild, under 5 cells/glomerulus), 2 (moderate, 5–9 cells/glomerulus), and 3 (severe, 10 and over cells/glomerulus). Interstitial CD68 ( +) cell infiltration was classified into four grades: 0 (none), 1 (mild, under 10 cells/field at × 200), 2 (moderate, 10–29 cells/field at × 200), and 3 (severe, 30 and over cells/field at × 200). There was no interstitial area with grade 0 CD68 staining (absent for macrophage infiltration) in all specimens (CD68 immunohistochemical stain, × 400 in upper row and × 200 in upper row; bar = 50 μm; N/A, not available)

Steroid treatment was performed in nine cases with CD-IgAN (50%) and nine cases with NOS-IgAN (91%). Table S3 details the number of cases for each hematuria and proteinuria grade before and after treatment in both groups described in Figure S3. Among cases with CD-IgAN, 22.2% retained a hematuria grade of 3 + , whereas 22.2% retained a proteinuria grade of 3 + even after treatment. However, none of the NOS-IgAN cases without CD had a hematuria or proteinuria grade of 3 + after steroid treatment (Fig. 3).

## Discussion

IBD may be complicated with IgAN [21, 22], with studies suggesting an association between intestinal immunity and the pathogenesis of IgAN. Therefore, the current study investigated the clinical and pathological differences between IgAN associated with IBD and general IgAN associated with upper respiratory tract inflammation, such as tonsillitis. The plasma cells of the upper respiratory tract mucosa primarily produces IgA1 [23]. However, the intestinal mucosa, especially Peyer’s patches, has been consider to predominantly secrete IgA2 (approximately 60% in mucosal cells) instead of IgA1 [24]. In the case of IgAN with CD, intestinal IgA2 may have been deposited in the glomerular mesangium and involved in the induction and progression of IgAN. However, our results indicated that among the deposited IgA subclasses, the IgA1 subclass was predominant in both the CD-IgAN and NOS-IgAN groups, with no significant difference in staining strength of IgA2 between both groups.

Thereafter, we investigated the deposition of Gd-IgA1, which has been reported to be specifically deposited in the glomeruli of primary IgAN [25]. Gd-IgA1 is an abnormal IgA1 of the IgA1 subclass that exhibits a structure in which galactose is deficient in the o-linked sugar chain at the hinge and N-acetyl galactosamine (GalNAC) is exposed [11, 12]. Notably, the current study found no difference in the degree of Gd-IgA1 deposition in the glomeruli regardless of CD complications, suggesting no significant difference between the CD-IgAN and NOS-IgAN groups in terms of the deposition of the Gd-IgA1 complex. In recent years, Gd-IgA1 has been detected in both secondary and primary IgAN, suggesting that secondary IgAN shares the same pathogenesis as primary IgAN. Additionally, negative views have emerged regarding the disease specificity of Gd-IgA1 [26].

After analyzing the histological findings of the kidney, the current study found that cases of CD-IgAN had significantly more severe global glomerulosclerosis, arteriolar hyalinosis grade, and IF/TA than those with NOS-IgAN. Comparing the Oxford classification scores revealed that the T score representing IF/TA was significantly higher in the CD-IgAN group than in the NOS-IgAN group. To confirm this tendency, we performed a meta-analytic comparison of MEST-C score using large-scale cohorts of IgAN patients reported in the previous literatures. The incidence of T1/2 was higher in our CD-IgAN group than in the large-scale IgAN cohorts reported by Barbour et al. from Canada [19] and Kamano et al. from Japan [20].

We speculate that these histological differences indicating advanced glomerulosclerosis and tubulointerstitial changes observed in the CD-IgAN patients were associated with the following three factors: (1) pathophysiology of CD (i.e., diarrhea and dehydration), (2) therapeutic agents for CD, and (3) systemic inflammation, including the intestinal tract.

First, during the course of IBD, dehydration due to diarrhea low dietary and water intake, surgery, etc. may reduce the circulating blood volume, causing tubular interstitial disorders and glomerulosclerosis [27]. Moreover, reports have showed that undernutrition and hypokalemia cause chronic tubular interstitial disorders. The reason for this is that reduced effective circulating blood volume may stimulate the renin–angiotensin–aldosterone system, followed by enhanced angiotensin II activation, causing arteriolar contraction, and glomerular ischemia, as well as interstitial fibrosis [28, 29]. In addition, evidence has shown that hyperuricemia, which is common in CD, may exacerbate glomerular sclerosis [30].

The second factor involves the effect of the therapeutics. Renal disorders associated with CD are present in 4–23% of patients with CD [31]. 5-ASA remains the main therapeutic agent for CD, with its renal adverse effects being collectively referred to as mesalamine-related kidney disease [32]. The mechanism through which mesalamine promotes renal damage appears to be through salicylate inhibition of the synthesis of intrarenal prostaglandins, which are vasoactive mediators of intrarenal blood flow and uncouple oxidative phosphorylation in mitochondria [27, 33]. Moreover, some reports have shown that mesalamine promotes renal damage histologically through interstitial nephritis [34], although similar findings having been reported in patients with CD not using 5-ASA [35]. Therefore, it is difficult to distinguish whether interstitial nephritis could be attributed to the drug or CD itself [31]. Given that 5-ASA agents were used in 12 of the 16 cases (75.0%, 2 cases not available) in this study, the advanced tubulointerstitial lesion may have been caused by the drug, although we could not precisely determine the etiology.

Third, the pathophysiology of CD itself, that is, immune abnormalities, may be involved. It is well known that macrophages and T cells produce large amounts of IL-23 and TNF-α in immune disorders, such as CD, and are considered to play a central role in the pathophysiology of CD [36]. These cytokines are also known to contribute to the exacerbation of tubulointerstitial lesions in IgAN [36]. Additionally, the mechanism by which dysfunctional macrophages promote intestinal fibrosis [37] has also been reported. In recent years, the mechanism and systemic response of B cell immune abnormalities [38, 39] have been clarified in CD [7]. B cell immune dysfunction has been reported to be involved in interstitial inflammation of chronic kidney disease, including IgAN [40], and immunological dysfunction of CD has been associated with IgAN from the viewpoint of immune dysfunction, which might act as an exacerbating factor for renal tubular interstitial disorders. Our result demonstrating the increased glomerular and interstitial macrophage infiltration in the CD-IgAN group than in the NOS-IgAN group suggests that some immunological abnormalities in CD may affect the macrophage infiltration in the kidney and promote glomerulosclerosis and interstitial fibrosis. Furthermore, in the pathology of IgAN, complement activity has been considered to promote glomerular sclerosis and interstitial fibrosis [41, 42]. In patients with CD, a previous study has shown that activated complement (mainly C3b) is strongly stained in the intestinal mucosa [43] and that the expression of complement C3 mRNA is increased in the resected ileocecal specimens [44]. Although we could not detect the difference of glomerular C3 staining intensity between CD-IgAN and NOS-IgAN, the effect of complement activation associated with CD may affect glomerular and tubulointerstitial inflammation of IgA nephropathy.

Factors considered to have caused the difference in therapeutic response to steroid therapy between the CD-IgAN and NOS-IgAN groups remain unclear. Regarding glomerulosclerosis and IF/TA, cases with IgAN who had more severe pathological changes were reported to be more resistant to steroid treatment than those with milder diseases [45]. The advanced IF/TA may be one of the factors influencing the poor therapeutic response in our CD-IgAN group; however, other factors, such as disease duration, effects of drugs, and immunological background, need to be further investigated to clarify the clinical features of IgAN complicated with CD.

The meta-analytic comparisons of MEST-C scores with two large-scale IgAN cohorts revealed several concerns related to the pathological relationship between CD and IgAN. One such concern is the lower incidence of segmental glomerulosclerosis, represented by the S score, in the CD-IgAN group than in the large-scale IgAN cohorts. We cannot speculate any inhibitory effects of CD on the formation of segmental sclerosis. As such, we believe that this tendency might have been an institutional bias on histological evaluation and the definition of segmental glomerulosclerosis in this study considering that the same tendency was observed in our NOS-IgAN group. Concerning the E and C scores, both our CD-IgAN and NOS-IgAN groups presented lower degrees of E and C compared to Kamano’s cohort but not Barbour’s cohort. Furthermore, it was our understanding that this could have been attributed to the institutional bias on histological evaluation of the E and C scores in Kamano’s study, as well as in the evaluation of the S score in the current study.

### Limitations of the present study

This study has several notable limitations. First, this was a retrospective case–control analysis performed at a single center with a relatively small sample size, especially in NOS-IgAN, and with a specialized bias for patients with IBD in our hospital. Second, the treatment protocol and treatment period were not standardized. Third, there may have been problems with the detection sensitivity of the immunohistological examinations using the formalin-fixed, paraffin-embedded section instead of the frozen section. Finally, in some cases, steroids were used for CD prior to IgAN treatment, and the effects of therapeutic drugs for CD on IgA disease activity and histological findings cannot be ruled out.

## Conclusions

IgA associated with CD did not differ from usual IgAN in terms of Gd-IgA1 staining and IgA subclasses in kidney biopsy specimens. These findings suggest no difference in the etiology between the CD-IgAN and NOS-IgAN groups. However, histological findings showed that patients with CD had severe glomerular sclerosis and IF/TA accompanying increased glomerular and interstitial macrophage infiltration and highly resistant clinical response to steroid treatment suggests that the immunological abnormality of CD may promote and activate the inflammatory processes of IgAN. It is necessary to accumulate further cases to clarify the relationship between CD and IgAN.

## Supplementary Information

Below is the link to the electronic supplementary material.

Supplementary file1 (PPTX 20541 KB)

Supplementary file2 (DOCX 37 KB)
