# Supplementary material for: Crohn’s disease may promote inflammation in IgA nephropathy: a case–control study of patients undergoing kidney biopsy
Source: Virchows Arch. 2022 Jul 9;481(4):553–63. doi: 10.1007/s00428-022-03373-w (PMC9534821; doi:10.1007/s00428-022-03373-w)
Supplement: Supplementary file 1 — Supplementary file1 (PPTX 20541 KB) [file 428_2022_3373_MOESM1_ESM.pptx]

## Slide 1
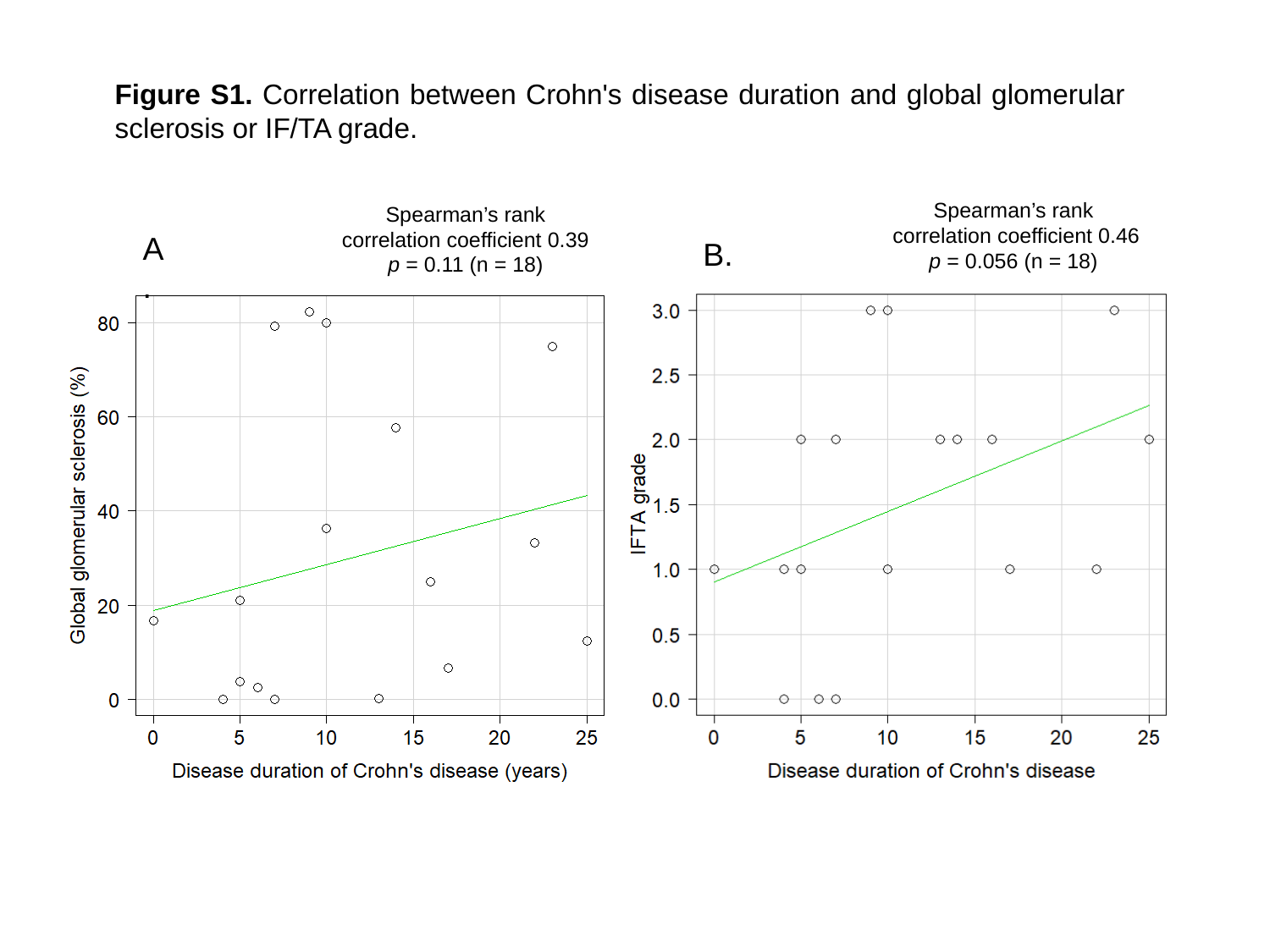

Figure S1. Correlation between Crohn's disease duration and global glomerular sclerosis or IF/TA grade.
Spearman’s rank correlation coefficient 0.39
p = 0.11 (n = 18)
Spearman’s rank
correlation coefficient 0.46
p = 0.056 (n = 18)
A.
B.

## Slide 2
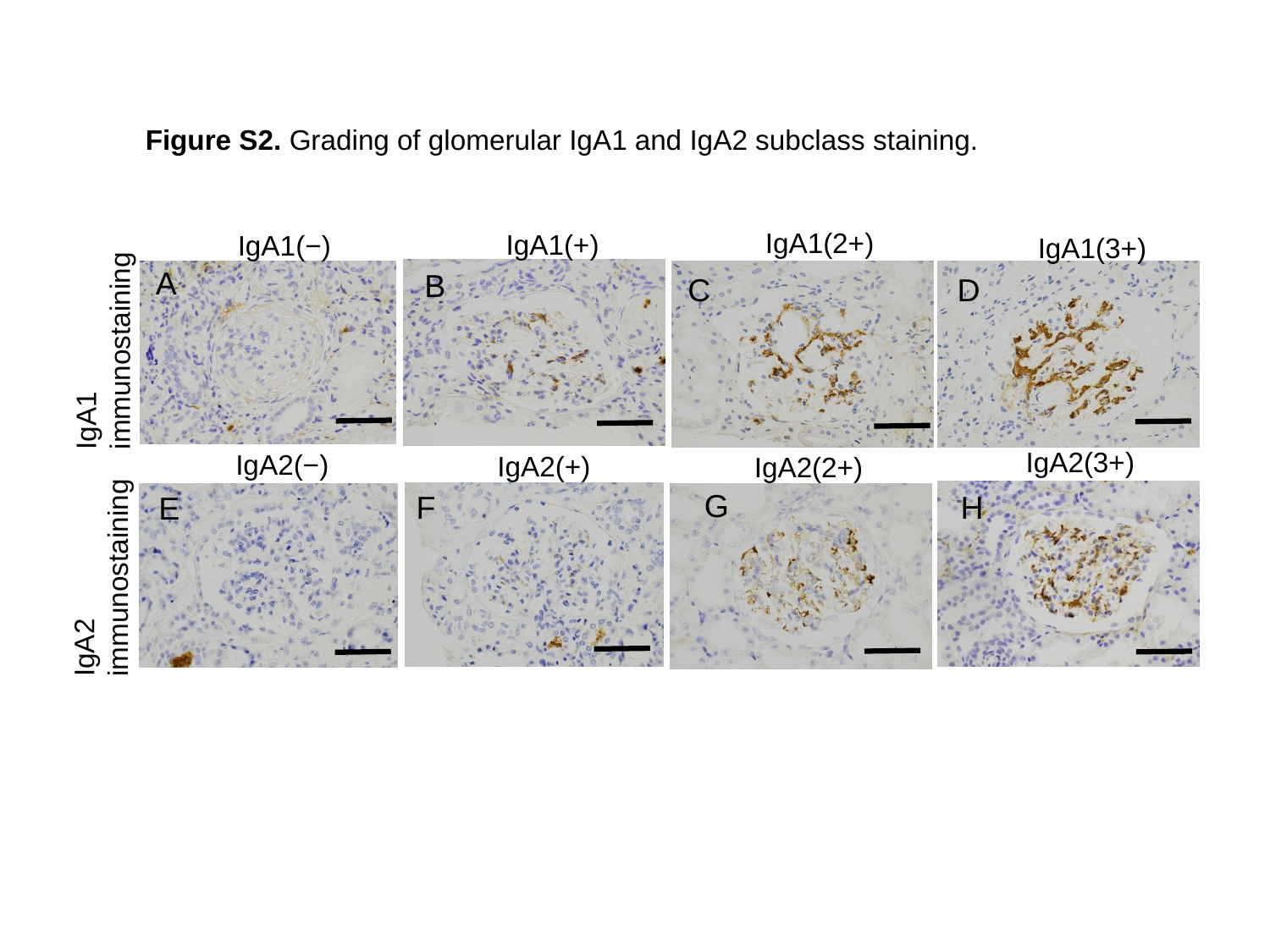

Figure S2. Grading of glomerular IgA1 and IgA2 subclass staining.
IgA1(2+)
IgA1(+)
IgA1(−)
IgA1(3+)
A
B
C
D
IgA1
immunostaining
IgA2(3+)
IgA2(−)
IgA2(+)
IgA2(2+)
G
F
H
E
IgA2 immunostaining

## Slide 3
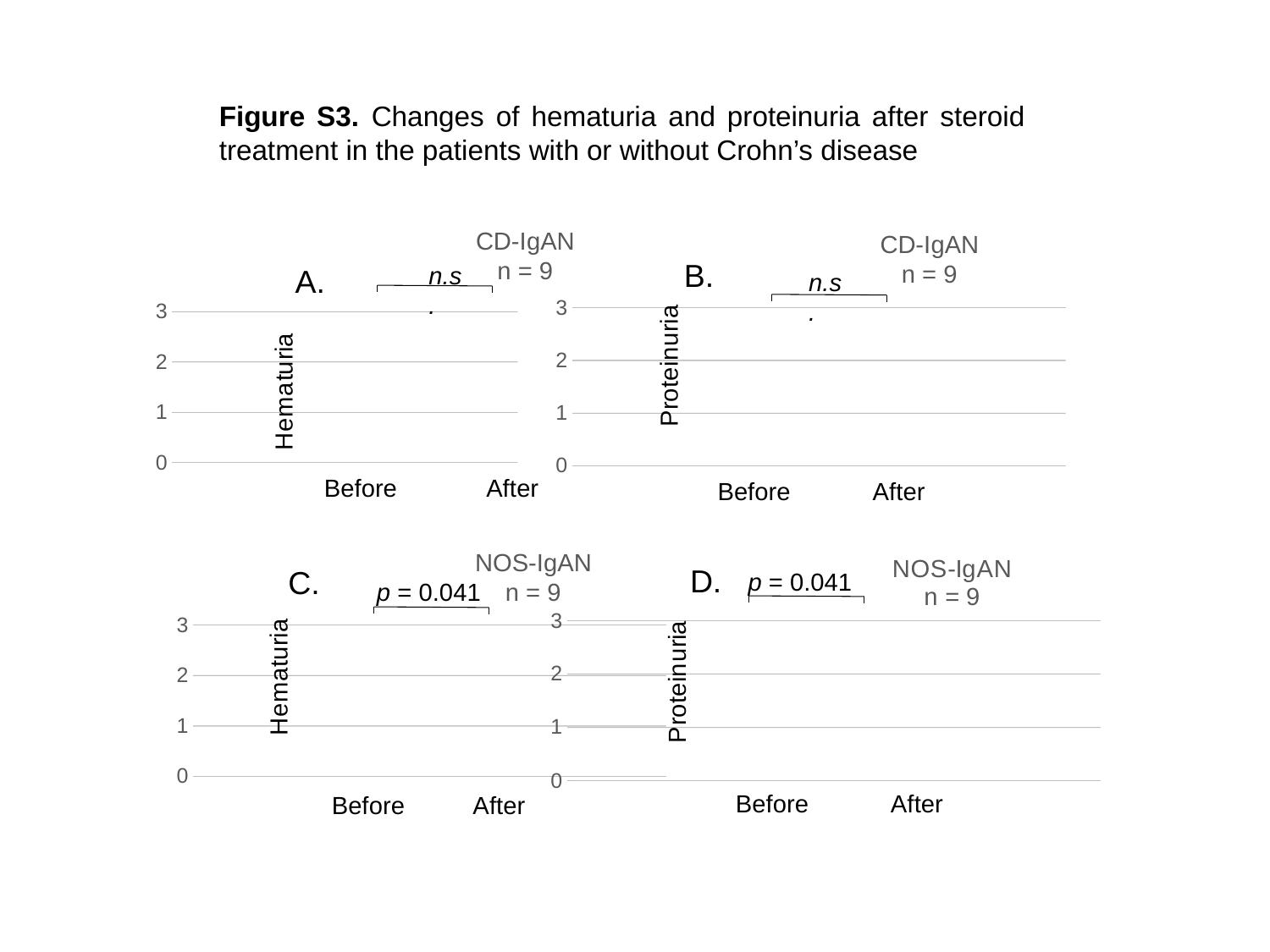

Figure S3. Changes of hematuria and proteinuria after steroid treatment in the patients with or without Crohn’s disease
CD-IgAN
n = 9
CD-IgAN
n = 9
B.
n.s.
A.
[unsupported chart]
[unsupported chart]
n.s.
Before After
Before After
NOS-IgAN
n = 9
[unsupported chart]
D.
C.
p = 0.041
[unsupported chart]
p = 0.041
Before After
Before After
