# Supplementary material for: Crohn’s disease may promote inflammation in IgA nephropathy: a case–control study of patients undergoing kidney biopsy
Source: Virchows Arch. 2022 Jul 9;481(4):553–63. doi: 10.1007/s00428-022-03373-w (PMC9534821; doi:10.1007/s00428-022-03373-w)
Supplement: Supplementary file 2 — Supplementary file2 (DOCX 37 KB) [file 428_2022_3373_MOESM2_ESM.docx]

**Table S1**. Comparison of clinical characteristics between IgA patients with and without Crohn’s disease.

| Characteristics | CD-IgAN  (n = 18) | NOS-IgAN  (n = 11) |  |
| --- | --- | --- | --- |
|  | mean ± s.d. | mean ± s.d. | *p-value* |
| Age (years) | 35.1 ± 12.2 | 34.7 ± 11.6 | NS (0.84) |
| Male gender, No (%) | 17 (94.4) | 6 (54.5) | 0.018* |
| Duration from onset to biopsy (years) | 2.7 ± 2.5 | 3.9 ± 5.1 | NS (0.80) |
| BMI (kg/m^2^) | 20.8 ± 3.0 | 23.8 ± 3.3 | 0.012* |
| Body weight (kg) | 60.6 ± 10 | 68.0 ± 14.1 | NS (0.17) |
| Systolic blood pressure (mmHg) | 122.4 ± 15.0 | 126.6 ± 14.2 | NS (0.37) |
| Use of RASI (yes) | 2 (11.1) | 1 (9.1) | NS (1.00) |
| Serum Cr level (mg/dL) | 1.5 ± 0.8 | 0.9 ± 0.3 | 0.003** |
| Serum IgA level (mg/dL) | 482 ± 220.0 | 400 ± 171.3 | NS (0.47) |
| Hematuria grade (pretreatment) (%) | 2.01 ± 1.18 | 1.72 ± 0.84 | NS (0.08) |
| (−) | 3 (16.7%) | 0 (0%) |  |
| (±) | 1 (5.6%) | 2 (18.2%) |  |
| (1+) | 1 (5.6%) | 2 (18.2%), |  |
| (2+) | 3 (16.7%) | 5 (45.5%) |  |
| (3+) | 10 (55.6%) | 2 (18.2%) |  |
| Proteinuria grade (pretreatment) (%) | 1.39 ± 1.09 | 1.45 ± 1.05 | NS (0.78) |
| (−) | 4 (22.2%) | 2 (18.2%) |  |
| (±) | 4 (22.2%) | 2 (18.2%) |  |
| (1+) | 0 (0.0%) | 1 (9.1%) |  |
| (2+) | 7 (38.9%) | 4 (36.4%) |  |
| (3+) | 3 (16.7%) | 2 (18.2%) |  |
| Treatment |  |  |  |
| Any type of steroid therapy | 9 (50.0%) | 9 (81.8%) | NS (0.13) |
| Oral steroid therapy, No. (%) | 9 (50.0%) | 9 (81.8%) | NS (0.13) |
| Steroid pulse therapy, No. (%) | 9 (50.0%) | 8 (72.7%) | NS (0.27) |
| Tonsillectomy, No. (%) | 7 (38.9%) | 6 (54.5%) | NS (0.47) |
| Use of 5-ASA, No. (%) | 12 (75.0%) ^a^ | - |  |

Mann–Whitney U test or Fisher’s test was used for statistical analysis. Abbreviations: No (%), number (%); BMI, body mass index; RASI, renin-angiotensin system inhibitor; Cr, creatinine; IgA, immunoglobulin A; 5-ASA, 5-aminosalicylic acid. ^a^The clinical records of 2 patients in the CD-IgAN group were not available for the use of 5-ASA; thus, the proportion of patients treated with 5-ASA was 75% (12/16 patients). **p* < 0.05, ***p* < 0.01. NS, not significant.

**Table S2**. Comparison of urinary findings at pre- and post-steroid treatment among patients in the CD-IgAN and NOS-IgAN groups

|  |  | CD-IgAN (n = 9) | | |  | NOS-IgAN (n = 9) | | |
| --- | --- | --- | --- | --- | --- | --- | --- | --- |
|  |  | Pre-  treatment | Post-treatment | *p-value* |  | Pre-treatment | Post-treatment | *p-value* |
|  |  |  |  |  |  |  |  |  |
| Hematuria | Mean ±s.d. | 2.78 ± 0.67 | 1.78 ± 0.97 | NS. |  | 1.56 ± 0.85 | 0.28 ± 0.67 | 0.041* |
|  | (−) | 0(0%) | 1(11.1%) |  |  | 0 (0%) | 7 (77.8%) |  |
|  | (±) | 0 (0%) | 0 (0.0%) |  |  | 2(22.2%) | 1 (11.1%) |  |
|  | (1+) | 1 (11.1%) | 2 (22.2%) |  |  | 2(22.2%) | 0 (0.0%) |  |
|  | (2+) | 0(0%) | 4 (44.4%) |  |  | 4(44.4%) | 1 (11.1%) |  |
|  | (3+) | 8 (88.9%) | 2 (22.2%) |  |  | 1(11.1%) | 0 (0.0%) |  |
|  |  |  |  |  |  |  |  |  |
| Proteinuria | Mean ±s.d. | 2.17 ± 0.79 | 2.11 ± 0.93 | NS. |  | 1.72 ± 1.03 | 0.22 ± 0.36 | 0.041* |
|  | (−) | 0(0.0%) | 1 (11.1%) |  |  | 1(11.1%) | 6 (66.7%) |  |
|  | (±) | 1(11.1%) | 1 (11.1%) |  |  | 0 (0.0%) | 2 (22.2%) |  |
|  | (1+) | 0 (0.0%) | 1 (11.1%) |  |  | 0 (0.0%) | 1 (11.1%) |  |
|  | (2+) | 5(55.6%) | 4 (44.4%) |  |  | 5 (55.6%) | 0 (0.0%) |  |
|  | (3+) | 3(33.3%) | 2 (22.2%) |  |  | 3 (33.3%) | 0 (0.0%) |  |

Mann–Whitney U test or Fisher’s test was used for statistical analysis. **p* < 0.05. NS, not significant.

**Table S3**. Comparison of MEST-C scores between CD-IgA group of this study and other two IgAN cohorts in the previous papers [19, 20].

|  |  | CD-IgAN group in this study (N=18) | | IgAN cohort of Barbour et al. (N=901)^19)^ |  | IgAN cohort of Kamano et al. (N=771) ^20)^ |  |
| --- | --- | --- | --- | --- | --- | --- | --- |
|  |  | N (%) |  | N (%) | *p-value* | N (%) | *p-value* |
| M | 0 | 10(55.56) |  | 518(57.49) | 1 | 525(68.09) | 0.31 |
|  | 1 | 8(44.44) |  | 383(42.51) |  | 246(31.91) |  |
| E | 0 | 17(94.44) |  | 738(81.90) | 0.22 | 499(64.72) | 0.0098** |
|  | 1 | 1(5.56) |  | 163(18.09) |  | 272(35.28) |  |
| S | 0 | 14(77.78) |  | 225(24.97) | ＜0.001** | 162(21.03) | <0.001** |
|  | 1 | 4(22.22) |  | 676(75.03) |  | 609(78.97) |  |
| T | 0 | 10(55.56) |  | 702(77.91) | 0.045* | 683(88.59) | <0.001** |
|  | 1 | 6(33.33) |  | 161(17.87) |  | 76(9.86) |  |
|  | 2 | 2(11.11) |  | 38(4.23) |  | 12(1.56) |  |
| C | 0 | 12(66.67) |  | 747(82.91) | 0.12 | 315(40.86) | 0.0497* |
|  | 1 | 6(33.33) |  | 154(17.09) |  | 456(59.14) |  |

Fisher’s test was used for statistical analysis. **p* < 0.05, ***p* < 0.01. NS, not significant.

**Table S4**. Comparison of MEST-C scores between NOS-IgA group of this study and other two IgAN cohorts in previous papers [19, 20].

|  |  | NOS-IgAN group in this study (N=11) | | IgAN cohort of Barbour et al. (N=901)^19)^ | |  | IgAN cohort of Kamano et al. (N=771) ^20)^ |  |
| --- | --- | --- | --- | --- | --- | --- | --- | --- |
|  |  | N (%) |  | | N (%) | *p-value* | N (%) | *p-value* |
| M | 0 | 9(81.82) |  | | 518(57.49) | 0.13 | 525(68.09) | 0.52 |
|  | 1 | 2(18.18) |  | | 383(42.51) |  | 246(31.91) |  |
| E | 0 | 11(100) |  | | 738(81.90) | 0.23 | 499(64.72) | 0.0106* |
|  | 1 | 0(0) |  | | 163(18.09) |  | 272(35.28) |  |
| S | 0 | 11(100) |  | | 225(24.97) | ＜0.001** | 162(21.03) | ＜0.001** |
|  | 1 | 0(0) |  | | 676(75.03) |  | 609(78.97) |  |
| T | 0 | 11(100) |  | | 702(77.91) | 0.34 | 683(88.59) | 0.674 |
|  | 1 | 0(0) |  | | 161(17.87) |  | 76(9.86) |  |
|  | 2 | 0(0) |  | | 38(4.23) |  | 12(1.56) |  |
| C | 0 | 9(81.82) |  | | 747(82.91) | 1 | 315(40.86) | 0.0101* |
|  | 1 | 2(18.18) |  | | 154(17.09) |  | 456(59.14) |  |

Fisher’s test was used for statistical analysis. **p* < 0.05, ***p* < 0.01. NS, not significant.
